# Prognostic Potential of Immune Inflammatory Biomarkers in Breast Cancer Patients Treated with Neoadjuvant Chemotherapy

**DOI:** 10.3390/cancers14215287

**Published:** 2022-10-27

**Authors:** Marta Truffi, Federico Sottotetti, Nadav Gafni, Sara Albasini, Francesca Piccotti, Carlo Morasso, Valentina Tibollo, Michela Mocchi, Valentina Zanella, Fabio Corsi

**Affiliations:** 1 Istituti Clinici Scientifici Maugeri IRCCS, 27100 Pavia, Italy; 2 Dipartimento di Scienze Biomediche e Cliniche, Università di Milano, 20157 Milano, Italy

**Keywords:** inflammatory biomarkers, breast cancer care, neoadjuvant chemotherapy, blood count, prognostic factors, tailored therapy

## Abstract

**Simple Summary:**

Systemic inflammation is associated with an increased aggressiveness of breast cancer and can contribute to a decreased activity of neoadjuvant treatments. Biomarkers of systemic inflammation are easily obtained from routine blood counts and are highly cost-effective, having great potential to steer cancer prognosis in clinical practice. In our study, we tested the hypothesis that high values of these biomarkers might have an effect on the clinical outcomes in a population of patients treated with neoadjuvant chemotherapy for breast cancer. The results of our study, together with data from the literature, hint at a possible role of inflammatory markers in the diagnostic and therapeutic algorithm of breast cancer, where specific pre-operative blood cell ratios could be used in combination with biological and clinical factors to tailor adjuvant therapy.

**Abstract:**

Immune inflammatory biomarkers are easily obtained and inexpensive blood-based parameters that recently showed prognostic and predictive value in many solid tumors. In this study, we aimed to investigate the role of these biomarkers in predicting distant relapse in breast cancer patients treated with neoadjuvant chemotherapy (NACT). All breast cancer patients who referred to our Breast Unit and underwent NACT were retrospectively reviewed. The pre-treatment neutrophil-to-lymphocyte ratio (NLR), platelet-to-lymphocyte ratio (PLR), monocyte-to-lymphocyte ratio (MLR), and pan-immune-inflammation value (PIV) were calculated from complete blood counts. The primary outcome was 5-year distant-metastasis-free survival (DMFS). In receiver operating characteristic analyses, the optimal cutoff values for the NLR, PLR, MLR, and PIV were determined at 2.25, 152.46, 0.25, and 438.68, respectively. High levels of the MLR, but not the NLR, PLR, or PIV, were associated with improved 5-year DMSF in the study population using both univariate (HR 0.52, *p* = 0.03) and multivariate analyses (HR, 0.44; *p* = 0.02). Our study showed that the MLR was a significant independent parameter affecting DMFS in breast cancer patients undergoing NACT. Prospective studies are required to confirm this finding and to define reliable cutoff values, thus leading the way for the clinical application of this biomarker.

## 1. Introduction

Systemic inflammatory markers were introduced to the oncological field for the first time in 2001 by Slovakian intensivist Roman Zahorec [1]. He conducted research on post-operative oncological patients in the intensive care unit and found that alterations in their differential blood counts correlated with clinical picture severity. He identified the ratios between absolute blood cell counts as systemic inflammatory markers. Such biomarkers are easily obtained and highly cost-effective, confirming their potential usefulness in clinical practice. Since then, multiple studies have established the role of these markers as prognostic or predictive factors in solid tumors, as well as in several inflammatory conditions [2,3,4].

In the case of breast cancer (BC), the platelet-to-lymphocyte ratio (PLR) [5,6], the monocyte-to-lymphocyte ratio (MLR) [7], and the neutrophil-to-lymphocyte ratio (NLR) [8] have been investigated as both prognostic and predictive factors. In particular, the NLR has been found to be an independent prognostic factor for survival in most adjuvant treatment studies [9,10]. A high NLR is generally associated with poor prognosis, as neutrophils have the potential to inhibit anti-tumor immunity by secreting a variety of inflammatory mediators and suppressing the cytotoxic activity of T cells [11,12]. However, the correlation between the biomarker and patient survival is much less consolidated in the neoadjuvant setting [13,14,15]. Many studies emphasize that a low pre-treatment NLR is associated with an increased pathologic complete response (pCR) rate [16], but this is particularly related to the tumor molecular subtype, and its correlation with long-term outcomes is still debated [8,15,17]. Moreover, despite the NLR being the most commonly analyzed parameter of systemic inflammation, biomarkers that take into account platelets and monocytes deserve attention, as they have shown potential relevance [18,19]. Recently, the pan-immune inflammation value (PIV) has been reported as a new comprehensive biomarker involving the neutrophil, platelet, monocyte, and lymphocyte counts, with the claim to perform better than single immune inflammatory indicators in predicting survival outcomes [20,21]. However, a current limitation to the everyday use of blood cell-based biomarkers is the fact that there are no homogenous cutoffs since studies conducted on diverse populations around the globe yield different reference values.

In a previous study, we explored the role of preoperative systemic inflammatory biomarkers in a cohort of early BC undergoing upfront surgery without indication for neoadjuvant chemotherapy (NACT) and found that they are independent predictors of disease recurrence in ER+ HER2- early BC [22]. In this study, we retrospectively analyzed a population of 217 BC patients treated with NACT and investigated the prognostic role of pre-treatment systemic inflammatory markers. Each individual biomarker was assessed in relation to oncological outcomes relevant to this setting, in particular to distant-metastasis-free survival (DMFS).

## 2. Materials and Methods

### 2.1. Patients Population

Patients with age >18 years, histopathological diagnosis of invasive BC at core biopsy, completion of NACT and subsequent surgery, and absence of metastasis at diagnosis were retrospectively included in this study from the prospective database (DataBreast) of EUSOMA-accredited Breast Unit of Istituti Clinici Scientifici Maugeri (Pavia, Italy). Patients with in situ lesions and patients who underwent breast surgery without any prior pharmacological treatment were excluded from the study. Patients received adjuvant treatments (radiotherapy, chemotherapy, biological therapy, hormonal therapy) according to the standard of care.

### 2.2. Blood Count and Data Collection

For all patients, clinical data (anamnesis, tumor characteristics, and therapy) were collected and updated on a yearly basis in DataBreast, for at least 5 years of follow-up. Laboratory data on different cell blood counts were exported from the hospital clinical electronic repositories. For each patient, pre-treatment blood counts obtained within the two weeks preceding NACT start were considered for the analyses.

### 2.3. Study Design and Endpoint

The pre-treatment NLR, PLR, MLR, and PIV were calculated as previously reported [20,21,22,23]. The primary endpoint of the study was the 5-year DMFS. The onset of distant metastasis was assessed using imaging (computed tomography and positron emission tomography). Each biomarker was analyzed separately to investigate any potential association with the patient survival outcome.

### 2.4. Statistical Analysis

Statistical significance was set at *p* < 0.05 (two-tailed). For software used and all statistical analyses on the NLR, MLR, PLR, and PIV concerning Cox proportional hazard regression model, Kaplan–Meier method, and time-dependent receiver operating characteristic (ROC) curves, refer to methods previously described [21]. NACT regimen, age at diagnosis, clinical tumor stage (cT) and node stage (cN), Ki67 at biopsy, biological portrait at biopsy, grade at biopsy, and histological type of the tumors at biopsy were selected a priori as relevant clinical variables to be included in the Cox multivariate analyses.

## 3. Results

### 3.1. Patient and Tumor Characteristics

A total of 217 BC patients were included in the study. The clinical–pathological features of the lesions included in the study are presented in Table 1. The mean age at diagnosis was 52 (±11) years, and 54.2% of women had reached menopause. In 59 cases (27.2%) a NACT regimen with anthracyclines/FEC (Type 1) was performed; a total of 63 breast lesions (29%) were treated by anthracyclines/FEC and taxanes (Type 2), and 82 patients (37.8%) received anthracyclines/FEC, taxanes, and anti-HER2 regimen (Type 3). Most of the cases at diagnosis were clinically cT2 stage (55.1%), grading II (63.9%), and node negative (cN0, 49.1%) tumors. Ductal and lobular tumors at core biopsy represented 84.8% and 13.8% of the lesions, respectively. The biomolecular subtype at biopsy was ER+ HER2- in 37.7% of the cases, ER+ HER2+ in 30.7%, ER- HER2- in 16.7%, and ER- HER2+ in 14.9%. After NACT, 49 breast lesions (23.0%) reached clinical complete response (cCR), as evaluated using clinical and imaging assessment, and 53.5% of patients underwent mastectomy during surgery; the remaining received breast conservative surgery. A total of 83 (38.3%) patients reached pCR at final histopathology. Supplementary Table S1 shows the pCR distribution among different tumor biological portraits; each patient received standard treatment, so results were consistent with reliable answers to the care. The mean follow-up time was 44 (±18) months, and during the 5-year period, distant metastases (DM) occurred in 47 cases (21.7%). Supplementary Figure S1 shows the Kaplan–Meier curves for 5-year DMFS in the study population.

### 3.2. Implication of Immune Inflammatory Biomarkers on Survival Outcome

For the whole series of patients, the optimal cutoff values of the baseline NLR, PLR, and MLR were calculated based on the analysis of the ROC curves for the prediction of metastasis occurrence (Table 2). The cutoff value of the pre-treatment PIV was also analyzed as a potential inflammation marker that integrates information from different blood cell populations.

**Figure 1:**
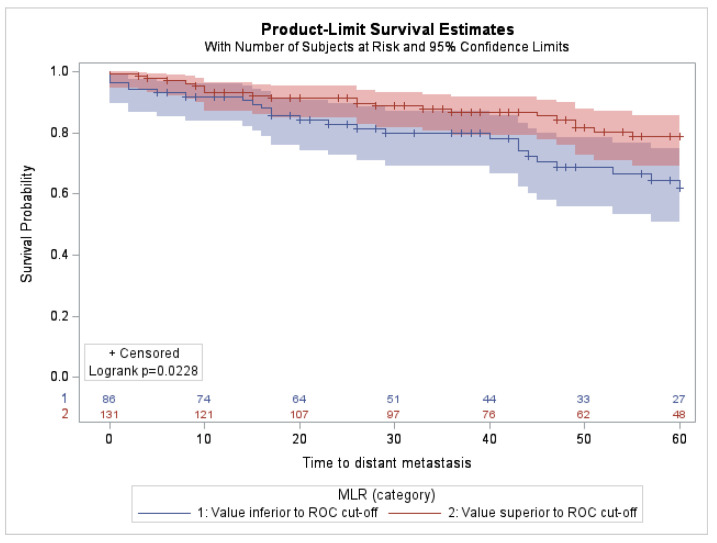
Kaplan–Meier curves for DMFS according to low vs. high MLRs in the study population (n = 217).

The univariate analysis of inflammatory markers, reported in Table 3, showed that NLR, PLR, and PIV were not associated with 5-year DMFS. (NLR: HR = 0.98; 95% CI, 0.54–1.75; *p*-value = 0.94. PLR: HR = 1.49; 95% CI, 0.84–2.67; *p*-value = 0.17. PIV: HR = 1.27; 95% CI, 0.70–2.28; *p*-value = 0.43.) By contrast, high MLR levels were significantly associated with improved DMFS (HR = 0.52; 95% CI, 0.29–0.92; *p*-value = 0.03). This association was also depicted by the Kaplan–Meier curves, showing that patients with an elevated MLR had a longer 5-year DMFS than those with a low MLR (Figure 1). The implication of the MLR on survival outcome did not depend on a specific biological portrait (Supplementary Figure S2) nor on the women’s menopausal state (*p*-value = 0.50).

### 3.3. Prediction Model According to Inflammatory Biomarkers

In order to further investigate whether pre-treatment systemic lymphocyte ratios represented reliable and informative prognostic biomarkers other than known clinical–pathologic features, a multivariate Cox analysis was performed taking into account blood-derived inflammatory markers together with patient age, cT, cN, Ki67 levels at core biopsy, tumor grade and histotype, tumor biological portrait, and NACT regimen. The results reported in Table 4 confirmed that among systemic inflammatory markers, the MLR was the only independent predictor of 5-year DMFS in the study population. High MLR values were significantly associated with improved 5-year DMFS (HR = 0.44; 95% CI, 0.22–0.86; *p*-value = 0.02), while the NLR, PLR, and PIV had no impact on survival outcome. Beyond the pre-treatment MLR, other independent risk factors for metastatic relapse were cT, cN, Ki67 status, tumor grade, and histological type at biopsy. We performed further multivariate analyses including all four variables, i.e.*,* NLR, MLR, PLR, and PIV, and clinical factors in order to verify if inflammatory biomarkers could affect each other; the results confirmed the MLR as an independent factor in 5-year DMFS (Supplementary Table S3) with an inferior hazard ratio (HR = 0.37 vs. HR = 0.44; Table 4) and 95% CIs totally stackable ([0.16–0.84] vs. [0.22–0.86]; Table 4).

## 4. Discussion

BC, one of the most common invasive cancer worldwide, is a complex disease often characterized by both biological (i.e., intrinsic to patient or disease biology) and non-biological (i.e., socioeconomic, cultural, environmental, and behavioral) factors [24]. Inflammation due to previous pathologic conditions or the immune response against the tumor is a key determinant of this complexity and is included among the fundamental hallmarks of cancer as defined by Hanahan and Weinberg in their seminal paper [25]. Systemic inflammation is associated with increased BC aggressiveness and can contribute to a decreased activity of NACT as demonstrated by a recent meta-analysis [16] and a retrospective analysis of two randomized studies that showed a correlation between high baseline inflammatory markers and a lower likelihood of achieving pCR [26].

In our study, we tested the hypothesis that high values of inflammatory systemic markers might have an effect on 5-year clinical outcomes in a population of patients treated with NACT for BC. We found that the pre-treatment MLR was an independent predictor of 5-year DMFS, while the NLR, PLR, and PIV were not significantly associated with the patient’s outcome.

Most of the analyzed patients carried adverse prognostic features such as HER2 overexpression or amplification (45.6%) or triple-negative phenotype (16.7%). Noticeably, not every HER2+ patient received trastuzumab as part of their neoadjuvant regimen, depending on the availability of the drug in Italy at the time of treatment. Despite this difference with current clinical practice standards, 38.3% of the global population obtained a pCR following systemic treatment, which is in line with reference data.

The results from our study in part contradict the currently available literature, according to which an elevated NLR has a detrimental effect on patient outcomes. This negative correlation is especially found among patients with Hormone Receptor-negative and/or HER2-negative BC; however, it is less clear among HER2+ patients [9,10,27]. The impact of MLR on BC prognosis has been less extensively studied. Some publications detected an association between a high MLR and worse BC prognosis [14,27], whereas other studies reported no correlations between MLR and oncological outcomes [14,28]. A recent retrospective paper by Tianien et al. suggested that a high MLR could have a detrimental role in terms of overall survival and BC-specific survival in early BC patients; however, high NLR and MLR correlated with poor survival among HER2+ patients only in the subgroup that did not receive adjuvant trastuzumab [7]. The authors hypothesized a potential role of trastuzumab in the modulation of host systemic inflammation that is able to overcome the poor prognostic impact of high baseline NLR and MLR in this setting.

Despite potentially being a simple and straightforward way for predicting patient prognosis, systemic inflammatory markers cannot be used in all clinical settings. This is because the general inflammatory state can be caused by enhanced granulopoiesis as well as other pathologic and constitutional conditions; hence, it cannot be considered a universal adverse sign for cancer progression. In our study, most of the analyzed markers had no significant correlations with prognosis. Moreover, in our analysis, pCR did not correlate with blood cell ratio alterations (*p*-value = 0.09; see Supplementary Table S2). This implies that the effect of NACT and/or the clinical/biological features of the tumor could compensate for the negative prognostic impact of systemic inflammation.

Data from the literature, together with the results of our study, hint at a possible role of inflammatory markers in the diagnostic and therapeutic algorithm of BC, where specific pre-operative blood cell ratios could be used in combination with biological and anatomical factors to tailor adjuvant therapy. Additional studies are needed to evaluate the role of systemic inflammatory markers as prognostic and predictive factors in the quickly evolving and complex treatment of early BC, especially when considering the incorporation of immunotherapy in the early phase of the disease.

The limitations of this study derived from several factors. The retrospective nature of our research allowed us to produce only hypothesis-generating findings. The sample size of our population was rather small, thus precluding a precise analysis of the outcomes with low occurrence rates and the evaluation of prognostic markers in specific subgroups of patients. Additionally, the evolution of the treatment guidelines has modified the NACT protocols throughout the years, reducing the generalizability of the results. For instance, at the beginning of the 2000s, the guidelines promoted the use of 5-fluorouracil as a component of the NACT regimen. About 16.3% of patients with HER2+ disease did not receive anti-HER2 therapy, since it was not available in Italy before 2005–2006. Because of the heterogeneity of NACT regimens among patients, we assessed each inflammatory biomarker before the initiation of therapy; further studies should be established to investigate post-NACT biomarkers in a selected cohort of BC patients undergoing uniform treatments. Another important limitation is the choice of different cutoffs for blood cell ratios among studies, which prevents an accurate comparison of the results.

Our study suggests that the MLR could be an easy, reproducible, and useful marker to predict the outcome of early BC treated with NACT. Not all subtypes of BC trigger systemic inflammation in the same way, and the same can be said for granulocytic and lymphocytic activation. There may be other variables that are related to systemic inflammation and can influence disease progression that should be considered in future research. For instance, there is evidence that ties the alteration in lymphocyte ratios and obesity with an increased risk of relapse and death from BC [29]. Future studies would also have to explore the role of a multimeric protein complex participating in inflammation (i.e., inflammasome) in the context of cancer-related inflammation. The inflammasome either triggers cellular death or enhances the inflammatory response by promoting the release of interleukins [30]. There is evidence that in murine BC models, the inflammasome also promotes tumor infiltration via myeloid-derived suppressor cells and tumor-associated macrophages, a possible prognostic factor [31], but additional research in humans is required in order to further elucidate its role in cancer relapse or progression.

## 5. Conclusions

Our work suggests that an elevated pre-treatment MLR could predict 5-year DMFS in BC patients treated with NACT. Due to the retrospective nature of the study and the limitations in its design, additional prospective research is needed, taking into account the complex link between local and systemic inflammation in human cancer as well as the modulatory effect of new cancer treatment on systemic inflammation.

## Figures and Tables

**Table 1:** Clinical and pathological characteristics of the study population (n = 217 breast lesions).

Variable	BC (n = 217)	Variable	BC (n = 217)
**Age at diagnosis**	52 ± 11 [27–80]	**Ki67 at biopsy**	
**Hormonal status**		≤14%	62 (29.0%)
Pre-menopause	99 (45.8%)	>14%	152 (71.0%)
Menopause	117 (54.2%)	**Breast cCR after NACT**	
**NACT regimen**		No	164 (77.0%)
Type 1	59 (27.2%)	Yes	49 (23.0%)
Type 2	63 (29.0%)	**Type of surgery**	
Type 3	82 (37.8%)	Conservative surgery	101 (46.5%)
Others	13 (6.0%)	Mastectomy	116 (53.5%)
**Clinical tumor stage (cT)**		**Axillary dissection**	
1	43 (19.9%)	No	41 (18.9%)
2	119 (55.1%)	Yes	176 (81.1%)
3	23 (10.7%)	**SLN biopsy**	
4	31 (14.3%)	No	140 (64.5%)
**Clinical node stage (cN)**		Yes	77 (35.5%)
0	106 (49.1%)	**Breast pCR**	
1	85 (39.3%)	No	134 (61.7%)
2	19 (8.8%)	Yes	83 (38.3%)
3	6 (2.8%)	**Radiotherapy**	
**Histological type at biopsy**		No	68 (31.3%)
Ductal	184 (84.8%)	Yes	149 (68.7%)
Lobular	30 (13.8%)	**Hormonal therapy**	
Unknown	3 (1.4%)	No	66 (30.4%)
**Multifocality**		Yes	151 (69.6%)
No	167 (77.0%)	**NLR**	3.3 ± 2.6 [0.9–17.1]
Yes	50 (23.0%)	**PLR**	179.1 ± 114.2 [43.5–974.5]
**Grading at biopsy**		**MLR**	0.3 ± 0.2 [0.1–1.6]
I	5 (2.3%)	**PIV**	474.4 ± 573.9 [46.3–4964.5]
II	136 (63.9%)	**Death**	
III	72 (33.8%)	No	200 (92.2%)
**Biological portrait at biopsy**		Yes	17 (7.8%)
ER+/HER2-	81 (37.7%)	**Follow-up (months)**	44 ± 18 [3–60]
ER+/HER2+	66 (30.7%)	**DM**	
ER-/HER2+	32 (14.9%)	No	170 (78.3%)
ER-/HER2-	36 (16.7%)	Yes	47 (21.7%)
**Progesteron receptor at biopsy**		**Time to DM (months)**	40 ± 20 [0–60]
Negative	87 (40.5%)		
Positive	128 (59.5%)		

**Table 2:** Optimal cutoff values of the pre-treatment NLR, PLR, MLR, and PIV based on ROC curve analysis for prediction of 5-year DMFS in the study population.

	NLR	PLR	MLR	PIV
**AUC**	0.50	0.55	0.57	0.47
**Cutoff**	2.25	152.46	0.25	438.68

**Table 3:** Univariate analysis of the pre-treatment NLR, PLR, MLR, and PIV systemic inflammatory biomarkers predictive of 5-year DMFS.

	NLR			PLR			MLR			PIV		
	HR	95% CI	*p*-Value	HR	95% CI	*p*-Value	HR	95% CI	*p*-Value	HR	95% CI	*p*-Value
Value inferior to ROC cutoff (low)	0.98	0.54–1.75	0.94	1.49	0.84–2.67	0.17	0.52	0.29–0.92	0.03	1.27	0.70–2.28	0.43
Value superior to ROC cutoff (high)	Ref.			Ref.			Ref.			Ref.		

**Table 4:** Multivariate analyses of inflammatory and clinical characteristics in relation to 5-year DMFS in the study population (n = 217).

Variable	HR	95% CI	*p*-Value	Variable	HR	95% CI	*p*-Value
NLR				MLR			
High	0.84	0.44–1.59	0.59	High	0.44	0.22–0.86	0.02
Low	Ref.			Low	Ref.		
**Age at diagnosis**	0.99	0.96–1.02	0.56	**Age at diagnosis**	0.99	0.96–1.02	0.42
**Clinical tumor stage (cT)**				**Clinical tumor stage (cT)**			
1	0.18	0.05–0.65	0.01	1	0.18	0.05–0.62	0.01
2	0.22	0.11–0.43	<0.0001	2	0.18	0.09–0.36	<0.0001
3-4	Ref.			3-4	Ref.		
**Clinical node stage (cN)**				**Clinical node stage (cN)**			
cN0	0.43	0.23–0.83	0.01	cN0	0.46	0.24–0.89	0.02
cN+	Ref.			cN+	Ref.		
**Ki67 at core biopsy**				**Ki67 at core biopsy**			
≤14%	0.59	0.29–1.22	0.15	≤14%	0.45	0.21–0.97	0.04
>14%	Ref.			>14%	Ref.		
**Grading at biopsy**				**Grading at biopsy**			
I-II	4.84	1.84–12.75	0.001	I-II	4.03	1.52–10.68	0.01
III	Ref.			III	Ref.		
**Histological type at biopsy**				**Histological type at biopsy**			
Ductal	0.73	0.31–1.74	0.48	Ductal	0.62	0.27–1.42	0.26
Lobular	Ref.			Lobular	Ref.		
**Biological portrait**				**Biological portrait**			
ER+/HER2-	0.47	0.21–1.06	0.07	ER+/HER2-	0.49	0.21–1.11	0.09
ER+/HER2+	0.28	0.08–1	0.05	ER+/HER2+	0.36	0.1–1.27	0.11
ER-/HER2+	0.85	0.16–4.58	0.85	ER-/HER2+	0.91	0.17–4.91	0.91
ER-/HER2-	Ref.			ER-/HER2-	Ref.		
**NACT regimen**				**NACT regimen**			
Type 1	0.73	0.15–3.6	0.70	Type 1	0.86	0.17–4.27	0.85
Type 2	1.44	0.31–6.72	0.64	Type 2	1.75	0.37–8.16	0.48
Type 3	0.58	0.09–3.92	0.57	Type 3	0.49	0.07–3.35	0.47
Others	Ref.			Others	Ref.		
**PLR**				**PIV**			
High	0.92	0.49–1.74	0.80	High	0.81	0.41–1.6	0.55
Low	Ref.			Low	Ref.		
**Age at diagnosis**	0.99	0.96–1.02	0.52	**Age at diagnosis**	0.99	0.96–1.02	0.52
**Clinical tumor stage (cT)**				**Clinical tumor stage (cT)**			
1	0.18	0.05–0.65	0.01	1	0.18	0.05–0.65	0.01
2	0.22	0.11–0.43	<0.0001	2	0.21	0.11–0.42	<0.0001
3-4	Ref.			3-4	Ref.		
**Clinical node stage (cN)**				**Clinical node stage (cN)**			
cN0	0.42	0.22–0.8	0.01	cN0	0.43	0.23–0.82	0.010
cN+	Ref.			cN+	Ref.		
**Ki67 at core biopsy**				**Ki67 at core biopsy**			
≤14%	0.59	0.29–1.22	0.15	≤14%	0.58	0.28–1.21	0.15
>14%	Ref.			>14%	Ref.		
**Grading at biopsy**				**Grading at biopsy**			
I-II	4.98	1.89–13.07	0.001	I-II	4.90	1.86–12.87	0.001
III	Ref.			III	Ref.		
**Histological type at biopsy**				**Histological type at biopsy**			
Ductal	0.68	0.3–1.56	0.36	Ductal	0.66	0.29–1.52	0.33
Lobular	Ref.			Lobular	Ref.		
**Biological portrait**				**Biological portrait**			
ER+/HER2-	0.46	0.2–1.05	0.07	ER+/HER2-	0.45	0.2–1.03	0.06
ER+/HER2+	0.28	0.08–0.97	0.04	ER+/HER2+	0.29	0.08–1.01	0.05
ER-/HER2+	0.84	0.15–4.59	0.84	ER-/HER2+	0.85	0.16–4.56	0.85
ER-/HER2-	Ref.			ER-/HER2-	Ref.		
**NACT regimen**				**NACT regimen**			
Type 1	0.73	0.15–3.63	0.70	Type 1	0.72	0.14–3.55	0.68
Type 2	1.47	0.31–6.91	0.63	Type 2	1.48	0.32–6.91	0.62
Type 3	0.60	0.09–4.04	0.60	Type 3	0.55	0.08–3.81	0.54
Others	Ref.			Others	Ref.		

## Data Availability

Raw data are available at 10.5281/zenodo.7225411.

## Supplementary Materials

The following supporting information can be downloaded at: https://www.mdpi.com/article/10.3390/cancers14215287/s1, Table S1: Incidence of pathological complete response (pCR) between different biological portraits, Table S2: Incidence of pathological complete response (pCR) between MLR categories, Table S3: Multivariate analysis of all inflammatory biomarkers (PIV, NLR, MLR, and PLR) and clinical characteristics in relation to 5-year DMFS in the study population (n = 217), Figure S1: Kaplan–Meier curves for 5-year DMFS in the study population (n = 217), Figure S2: Kaplan–Meier curves for 5-year DMFS according to low vs. high MLRs in patients with ER+ HER2- (A), ER+ HER2+ (B), ER- HER2+ (C), and ER- HER2- (D) BC.
