# Supplementary material for: Prognostic Potential of Immune Inflammatory Biomarkers in Breast Cancer Patients Treated with Neoadjuvant Chemotherapy
Source: Cancers (Basel). 2022 Oct 27;14(21):5287. doi: 10.3390/cancers14215287 (PMC9658892; doi:10.3390/cancers14215287)

Supplementary Materials

Table S1: Rate of pathological complete response (pCR) in different biological portraits of BC.

Biological portrait	Breast pCR	
	No	Yes
ER+/HER2-	71 (87.7%)	10 (12.3%)
ER+/HER2+	31 (47%)	35 (53%)
ER-/HER2+	7 (21.9%)	25 (78.1%)
ER-/HER2-	24 (66.7%)	12 (33.3%)

Table S2: Incidence of pathological complete response (pCR) between MLR categories.

	Breast pCR		
	No	Yes	
MLR			p-value
Low	59 (68.6%)	27 (31.4%)	0.09
High	75 (57.2%)	56 (42.8%)	

Table S3: Multivariate analysis including all 4 systemic Inflammatory biomarkers (NLR, MLR, PLR, PIV) and clinical factors in relation to 5-years DMFS in the study population (n=217).

Variables	DMFS		
	HR	95%CI	p
**PIV**			
High	1.33	0.55-3.22	0.53
Low	Ref.		
**NLR**			
High	1.00	0.47-2.12	1.00
Low	Ref.		
**PLR**			
High	1.12	0.56-2.24	0.74
Low	Ref.		
**MLR**			
High	0.37	0.16-0.84	0.02
Low	Ref.		
**Age at diagnosis**	0.99	0.96-1.02	0.42
**cT**			
cT1	0.18	0.05-0.63	0.007
cT2	0.19	0.10-0.39	<0.0001
cT3-4	Ref.		
**cN**			
cN0	0.47	0.24-0.91	0.02
cN+	Ref.		
**Ki67 at core biopsy**			
≤14%	0.43	0.20-0.93	0.03
>14%	Ref.		
**Grading at biopsy**			
I-II	3.93	1.47-10.48	0.006
III	Ref.		
**Histological type at biopsy**			
Ductal	0.63	0.25-1.54	0.31
Lobular	Ref.		
**Biological portrait**			
ER+/Her2–	0.54	0.23-1.27	0.16
ER+/Her2+	0.36	0.10-1.28	0.11
ER-/Her2+	0.95	0.17-5.33	0.96
ER-/Her2 -	Ref.		
**NACT regimen**			
Type 1	0.86	0.17-4.28	0.86
Type 2	1.62	0.34-7.66	0.54
Type 3	0.53	0.08-3.65	0.52
Type 4	Ref.		

Figure S1: Kaplan-Meier curves for 5-years DMFS in the study population (n=217).

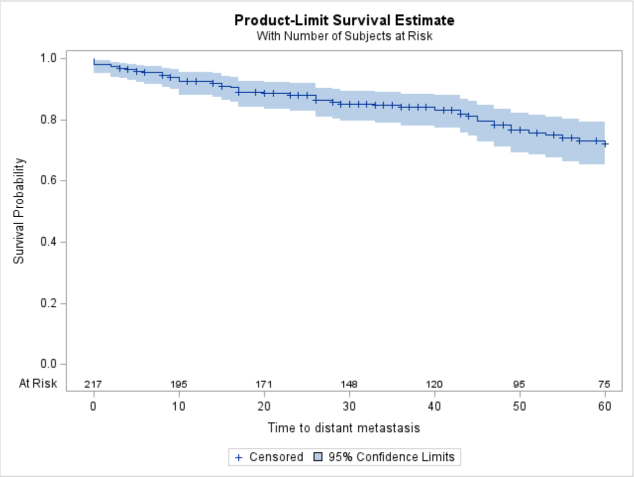

Figure S2: Kaplan-Meier curves for 5-years DMFS according to low vs. high MLR in patients with ER+ HER2- (A), ER+ HER2+ (B), ER- HER2+ (C), ER- HER2- (D) BC.

A.

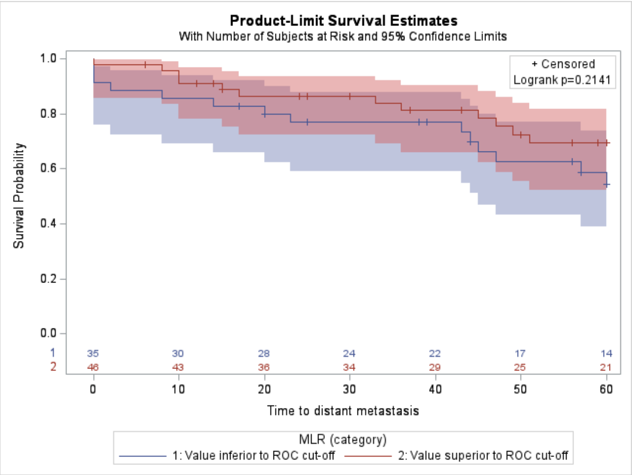

B.

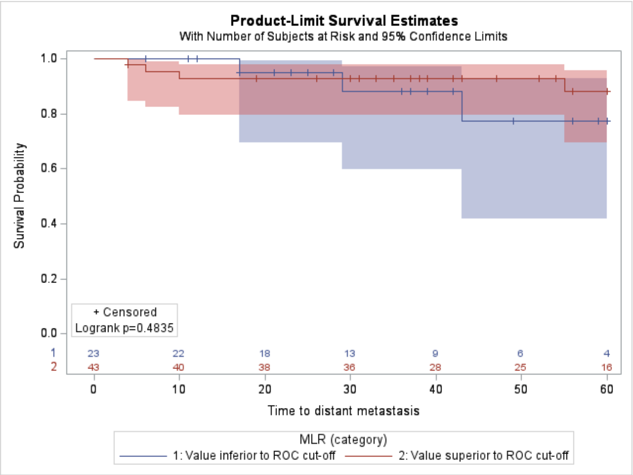

C.

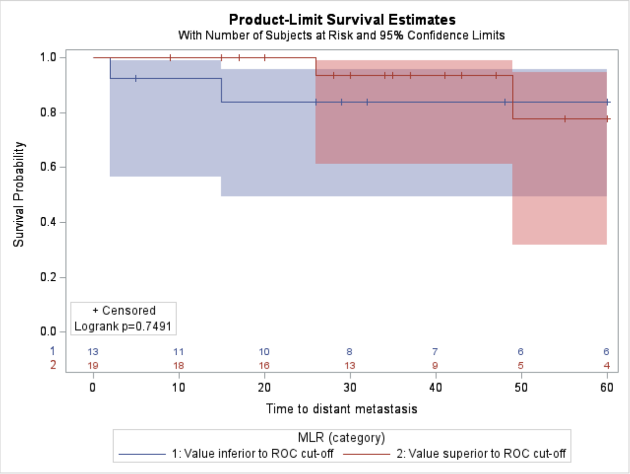

D.

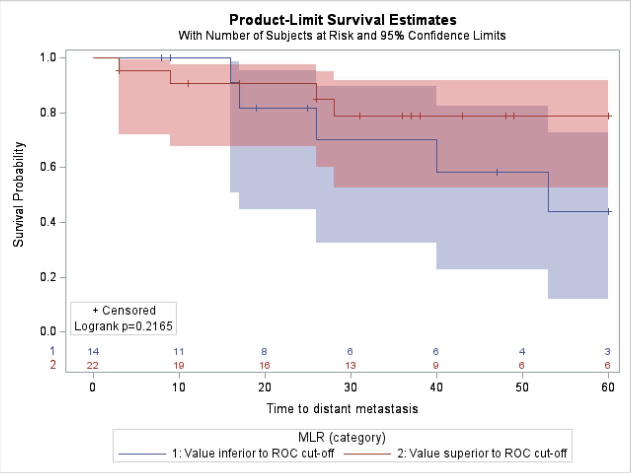

Supplement: Supplementary file 1 [file cancers-14-05287-s001.zip › cancers-1942862-supplementary.pdf]
